# Consensus treatment plans for periodic fever, aphthous stomatitis, pharyngitis and adenitis syndrome (PFAPA): a framework to evaluate treatment responses from the childhood arthritis and rheumatology research alliance (CARRA) PFAPA work group

**DOI:** 10.1186/s12969-020-00424-x

**Published:** 2020-04-15

**Authors:** Gil Amarilyo, Deborah Rothman, Kalpana Manthiram, Kathryn M. Edwards, Suzanne C. Li, Gary S. Marshall, Cagri Yildirim-Toruner, Kathleen Haines, Polly J. Ferguson, Geraldina Lionetti, Julie Cherian, Yongdong Zhao, Patricia DeLaMora, Grant Syverson, Simona Nativ, Marinka Twilt, Ian C. Michelow, Yuriy Stepanovskiy, Akaluck Thatayatikom, Liora Harel, Shoghik Akoghlanian, Lori Tucker, Mariana Correia Marques, Hemalatha Srinivasalu, Evan J. Propst, Greg R. Licameli, Fatma Dedeoglu, Sivia Lapidus, for the CARRA PFAPA Consensus Treatment Plan Workgroup, Ronald Laxer, Lisa Imundo, Paul Tsoukas, Peter Wright, Kelly Brown, Rima Khasawneh, Rosie Scuccimarri, Evan Mulvihill, Meghan Aabo, Edwin Anderson, Leslie Abramson, Daniela Adelean, Danielle Dumez, Marla Guzman, Renee Pang, Ellen Go, Katalin Koranyi, Donald Goldsmith, Hanna Kim, Andrew Zeft, Rayfel Schnieder, Victoria Statler, Lauren Steele, Lori Broderick, Hal Hoffman, Sriharsha Cherukumilli Grevich, Elizabeth Chalom, Michal Cidon, Robert Sundel, Nadine Saad, Deborah McCurdy, Grant Schulert, Ali Yalcindag, Eric Yen, Sara Stern, Karen Durrant, Yonatan Butbul, Jonathan Hausmann

**Affiliations:** 1 Pediatric Rheumatology Unit, Schneider Children’s Medical Center of Israel, Petach Tikva; Sackler Faculty of Medicine, Tel Aviv University, Tel Aviv, Israel; 2 Massacusetts General Hospital for Children, Boston, MA USA; 3 National Human Genome Research Institute, National Institutes of Health, Bethesda, MD USA; 4 Vanderbilt University School of Medicine, Nashville, TN USA; 5 Joseph M Sanzari Children’s Hospital, Hackensack Meridian Health, Hackensack, NJ USA; 6 Department of Pediatrics, University of Louisville, Louisville, KY USA; 7 Nationwide Children’s Hospital, Columbus, OH USA; 8 Stead Family Department of Pediatrics, University of Iowa Carver College of Medicine, Iowa City, IA USA; 9 UCSF Benioff Children’s Hospital Oakland, Oakland, CA USA; 10 Stony Brook University Hospital, Stony Brook, NY USA; 11 Seattle Children’s Hospital, University of Washington, Seattle, WA USA; 12 Weill Cornell Medical College, New York, NY USA; 13 Medical College of Wisconsin, Wauwatosa, WI USA; 14 Goryeb Children’s Hospital, Morristown, NJ USA; 15 Rheumatology, Department of Pediatrics, Alberta Children’s Hospital, University of Calgary, Calgary, Alberta Canada; 16 Alpert Medical School of Brown University, Providence, RI USA; 17 Shupyk National Medical Academy of Postgraduate Education, Kyiv, Ukraine; 18 Department of Pediatrics, University of Florida, Gainesville, FL USA; 19 BC Children’s Hospital, Vancouver, BC Canada; 20 Boston Children’s Hospital, Harvard Medical School, Boston, MA USA; 21 Children’s National Medical Center, Washington, DC USA; 22 Hospital for Sick Children, University of Toronto, Toronto, ON Canada

**Keywords:** PFAPA, Periodic fever, Recurrent fever, Consensus treatment plan

## Abstract

**Background:**

Periodic fever, aphthous stomatitis, pharyngitis, and cervical adenitis (PFAPA) syndrome is the most common periodic fever syndrome in children. There is considerable heterogeneity in management strategies and a lack of evidence-based treatment guidelines. Consensus treatment plans (CTPs) are standardized treatment regimens that are derived based upon best available evidence and current treatment practices that are a way to enable comparative effectiveness studies to identify optimal therapy and are less costly to execute than randomized, double blind placebo controlled trials. The purpose of this project was to develop CTPs and response criteria for PFAPA.

**Methods:**

The CARRA PFAPA Working Group is composed of pediatric rheumatologists, infectious disease specialists, allergists/immunologists and otolaryngologists. An extensive literature review was conducted followed by a survey to assess physician practice patterns. This was followed by virtual and in-person meetings between 2014 and 2018. Nominal group technique (NGT) was employed to develop CTPs, as well as inclusion criteria for entry into future treatment studies, and response criteria. Consensus required 80% agreement.

**Results:**

The PFAPA working group developed CTPs resulting in 4 different treatment arms: 1. Antipyretic, 2. Abortive (corticosteroids), 3. Prophylaxis (colchicine or cimetidine) and 4. Surgical (tonsillectomy). Consensus was obtained among CARRA members for those defining patient characteristics who qualify for participation in the CTP PFAPA study.

**Conclusion:**

The goal is for the CTPs developed by our group to lead to future comparative effectiveness studies that will generate evidence-driven therapeutic guidelines for this periodic inflammatory disease.

## Background

Periodic fever, aphthous stomatitis, pharyngitis and cervical adenitis syndrome (PFAPA) is the most common periodic fever condition in children. The true prevalence and etiology remain unknown although one Scandinavian study estimated the incidence to be 2.3 per 10,000 children [1]. The diagnosis is often delayed, and treatment approaches vary [2–10]. Patients present in the preschool years with recurrent episodes of high spiking fevers lasting 3 to 7 days and occurring every 2 to 8 weeks [2–10]. Associated features during the febrile episodes include pharyngitis, cervical lymphadenopathy and/or aphthous stomatitis. PFAPA usually resolves after several years [1, 3, 5, 8–10]. However, the episodes impact quality of life in both affected children and their families.

Current treatment for PFAPA includes corticosteroids given at the onset of an episode, daily cimetidine or colchicine, and tonsillectomy [2, 3]. However, there is no standard of care for PFAPA due to a lack of clinical trials.

Consensus treatment plans (CTPs) are a relatively new research methodology intended to reduce variation in treatment approaches. This process has been used for other rare diseases such as polyarticular juvenile idiopathic arthritis [11], systemic-onset juvenile idiopathic arthritis [12], juvenile dermatomyositis [13], juvenile localized scleroderma [14], chronic nonbacterial osteomyelitis [15] and juvenile lupus nephritis [16]. Therapies in use are initially identified through a literature review and physician surveys of current practice strategies. Nominal Group Technique (NGT) is applied to develop standardized CTPs based upon collected data. Major treatment regimens are defined and standardized for the disease in question [17]. Then, in prospective observational studies (after receiving institutional review board approval and obtaining parental permission with patients’ assent if applicable), physicians and patients, with their families, select the CTP they prefer, with response assessed based on agreed upon standardized outcome measures. Although this method is not randomized or blinded, it captures the response to therapy of a larger group of patients, typical of those seen in routine practice, prospectively, in a less costly way than double blind, randomized controlled trials. This is a major advantage for the study of rare diseases for which high-quality evidence-based data are lacking [17].

## Methods

CARRA was established to conduct collaborative research to prevent, treat, and cure childhood rheumatic diseases. The CARRA PFAPA Working Group is composed of North American, European, and Israeli pediatric rheumatologists, who are members of CARRA, as well as pediatric infectious disease specialists, otolaryngologists and allergists/immunologists with expertise in PFAPA. The group met via monthly teleconferences and at face-to-face meetings at the annual CARRA meeting from 2014 to 2018.

A search was conducted in 2015 for medical literature published between 1987 to 2014 and later expanded to 2018, using PubMed with the following medical subject headings (MeSH terms): (PFAPA OR (periodic fever AND aphthous stomatitis) OR (periodic fever AND pharyngitis)) AND (pediatric OR pediatrics OR paediatric OR paediatrics OR child OR children OR adolescent OR adolescents OR infant OR infants)) AND (treatment OR colchicine OR cimetidine OR (glucocorticoids OR corticosteroids OR steroids) OR (anakinra OR interleukin 1) OR tonsillectomy). With date and English language filters applied to this search, there were 140 citations. When treatment options were included in the search, 88 publications resulted of which 25 fulfilled selection criteria. Individual case reports and reviews were excluded. Due to the limited number of randomized controlled studies, case series, and prospective observational studies were also included. Levels of evidence were graded from 1 to 4 according to guidelines established by the Oxford Centre for Evidence-Based Medicine. CEBM; online at https://www.cebm.net/index.aspx?o=5653 (Supplementary Table 1).

The initial literature review (1987–2014) informed the development of a survey that was sent to all members of CARRA and pediatric infectious disease specialists (members of the Pediatric Infectious Disease Society (PIDS)) and completed by 123 CARRA members and 154 PIDS members, which included clinical vignettes. The purpose was to learn how physicians diagnose PFAPA and to identify the most common treatments used [18].

Draft CTPs were generated by the PFAPA subcommittee using data derived from the survey, the literature review and standardized case definitions. NGT was used in which the moderator presented key questions, 1 to 2 min of discussion was permitted, and members voted with a required 80% agreement at CARRA Annual Scientific Meetings.

For determining response criteria and outcome measures, a literature search was conducted for diseases with similar manifestations such as the hereditary auto-inflammatory disorders, systemic-onset juvenile idiopathic arthritis and infectious pharyngitis. Since these response criteria had to be developed a priori, the search was focused on studies that included outcomes, response criteria, and quality of life measures to determine if they were relevant to PFAPA. This was followed by group discussion with the most relevant measures selected as a part of the NGT, presented at the PFAPA study group session held during the November 2017 American College of Rheumatology/Association of Rheumatology Health Professionals Annual Meeting.

Finally, a survey was sent to 100 CARRA members selected at random, of whom 90% expressed willingness to follow at least one of the treatment arms. This project was reviewed and approved by the CARRA CTP Advisory Committee, a group within CARRA that ensures that the development of CTPs has been done according to CARRA regulations.

## Results

The PFAPA Work Group defined patient inclusion characteristics (Table 1) that were slightly modified from the original proposed diagnostic criteria for PFAPA [4–6, 18]. First, the duration of fever attacks was defined as 3–7 days to avoid confusion with other autoinflammatory syndromes that may share some features with PFAPA but differ in their duration of febrile attacks, such as Familial Mediterranean Fever [19, 20]. Second, to exclude recurrent infections and malignancy, patients had to have had more than 6 stereotypical periodic febrile episodes. Exclusion of cyclic neutropenia is kept. Third, to ensure periodicity, these episodes had to occur with regularity, defined as a maximum variability of 1 week for fever cycles occurring at 2–4-week intervals and a maximum variability of 2 weeks for fever cycles occurring at 5–8-week intervals. Fourth, age of onset ≤5 years was omitted because only one-third of the physicians who responded to the preliminary survery [18] considered young age mandatory for the diagnosis of PFAPA.^5,^

**Table 1:** CTPs inclusion/exclusion criteria for patients with PFAPA

Patient Characteristics (All criteria must be fulfilled) • 3–7 days of fever (minimum fever of 102.2 °F (39 °C) for 3 days per episode) • ≥ 6 episodes occurring with regularity • Definition of Regularity: • If fever cycle is 2–4 weeks, 1 week of variability would be allowed. If fever cycle is 5–8 weeks, 2 weeks of variability will be allowed. • Should have associated pharyngitis. In the absence of pharyngitis, patient must have both aphthous stomatitis and cervical adenopathy. • Normal growth and development • Steroid Responsive (if corticosteroids are administered with an episode) - Definition of steroid responsiveness - patient will be considered steroid responsive if fever resolves within 24 h after a maximum steroid dose of 2 mg/kg (max 60 mg) given in a single dose or divided over 2 doses. Patients should NOT have • Other known autoimmune/autoinflammatory disorder • Immunodeficiency (e.g cyclic neutropenia) • Malignancy • Infection	

**Fig. 1:**
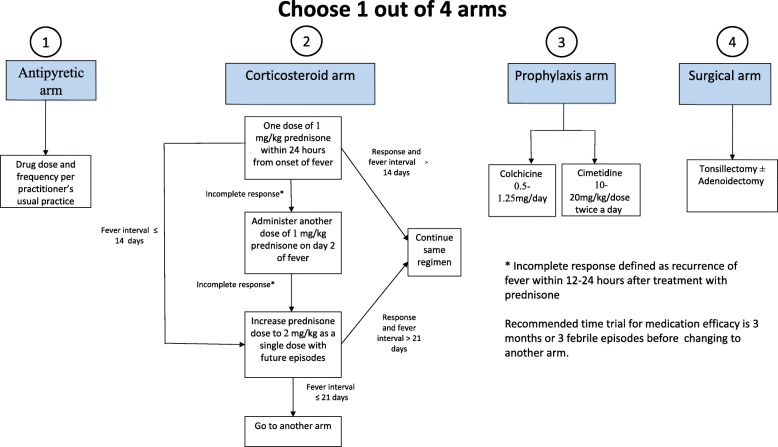
Consensus treatment plans for PFAPA

Four treatment strategies were defined based on the literature review, survey, and the process as described above:

1) Antipyretics during episodes; 2) Abortive treatment with corticosteroids; 3) Prophylaxis with colchicine or cimetidine; and 4) Surgical management with tonsillectomy (Fig. 1).

To evaluate efficacy, outcome measures were defined by the Work Group (Table 2) using NGT. The primary outcome was resolution of fever, categorized as complete (no fevers for 3 months), partial (reduced total number of days with fever over a period of 3 months), and no response (no change or increase in total days of fever over a period of 3 months). The group agreed that a particular regimen should be trialed for 3 febrile episodes to determine efficacy and, if deemed not effective by the physician and/or family, changed to another arm. Quality of life will be measured by missed school days and parental global assessment using a visual analog scale (a unidimensional measure of a characteristic or attitude that cannot easily be directly measured and is frequently used in populations with rheumatic diseases).

**Table 2:** CTPs Response Criteria

• Primary outcome: Fever (Measured when enrolled in CTP and every 3 months) A. Complete response: For antipyretic or steroid arm: complete resolution of fever for the particular flare within 12 h after medication administration for 3 months For prophylaxis or surgery arm: no fever for 3 months B. Partial response: Reduced number of days of fever over 3 months C. No response: No change or increased number of days of fever over 3 months • Other measures to be captured: 1) A decrease in the number of missed work days for the parent due to a PFAPA episode 2) Parent global score assessment on visual analog scale (VAS)	

## Discussion

This study is an international collaborative effort of the pediatric subspecialists (rheumatology, immunology, ENT and infectious diseases) with expertise in PFAPA to devise standardized treatment plans for this disease in the pediatric population. We describe the process of development of the CTPs which resulted in 4 treatment arms: antipyretic, abortive, prophylaxis, and surgical.

The four treatment arms presented in these CTP’s arms are the most studied arms in the literature [2, 3, 7–10] and were most frequently used by the physicians in the survey conducted by our group [18]. Specifically, our survey found that corticosteroids were rated as “effective” or “very effective” by 95%, tonsillectomy by 68%, antypirectics by 29% and cimetidine or colchicine by 19%. There are, however, some retrospective studies reports suggesting that cimetidine and colchicine may prevent PFAPA flares in about 50% of children [20–22].

Although the antipyretic arm was originally considered a control arm, antipyretics may be effective therapy for some children and are a reasonable option, especially for parents who are reluctant to use daily medication, are concerned about steroid side effects, or are concerned about the risks of tonsillectomy. This strategy was used prior to the discovery of effective abortive/prophylactic treatments, and no sequelae were reported in these patients.

The abortive arm was included to examine the efficacy of corticosteroids in halting the episode at onset. The corticosteroid regimen starts with the suggested dose of 1 mg/kg (max 60 mg), or 2 mg/kg (max 60 mg) in cases of inadequate response or shortened interval (≤14 days) between episodes. The dose was determined by the survey data which showed 64% of physicians used 1 mg/kg of prednisone (or prednisolone) and 29% used 2 mg/kg. Given both the adverse effects of corticosteroids and the survey showing preference for the 1 mg/kg dose [18], we chose to start with the lower dose, which can be increased to 2 mg/kg (max 60 mg) as a single dose if the response to 1 mg/kg was incomplete [9, 18]. We allowed intervals of ≥21 days for response to corticosteroids and recommended changing to another arm in the case of frequent flares (≤14 days). In the case of intervals between 14 and 21 days, it was recommended that the steroid dose be increased from 1 mg/kg to 2 mg/kg. If the interval increased to ≥21 days, then steroids would be continued at this higher dose. If they did not, then a different treatment arm is recommended.

The prophylaxis arms were intended to examine the efficacy of cimetidine and colchicine, although there is limited evidence in the literature on their effectiveness [18, 21, 22]. While other drugs were candidates for prophylaxis (e.g. montelukast) [23], only publications in peer-reviewed journals were included. Prophylaxis could be chosen by the physician as a first choice or after a failure of another arm.

There were a few small studies reporting a correlation between vitamin D insufficiency/deficiency [24] and PFAPA and one which found benefit of vitamin supplementation [25]. However, while promising; this preliminary evidence was not considered sufficient to suggest Vitamin D as prophylactic therapy for PFAPA.

Tonsillectomy has been reported to prevent recurrences and while this may be effective for patients with PFAPA, it is not without risk [26–28]. Although two small randomized control trials [26, 27] suggested that patients with PFAPA have less fever and less severe episodes after tonsillectomy compared to those receiving no surgery, a Cochrane review [28] concluded that this evidence is of moderate quality (meaning that further research is likely to have an important impact on our confidence in the estimate of effect) due to the small numbers of patients in the studies and concerns about the generalizability of the results. Moreover, the number of patients randomly allocated to surgery was too small to detect potentially important complications. Currently there are no data on the efficacy of tonsillotomy in PFAPA and therefore it was not included in the CTP.

Evidence for IL-1 blockade, while promising, was anecdotal or based on small case series and uncontrolled trials so was not included as a therapeutic option [8, 9, 29, 30].

For treatment arms, the benefits need to be carefully weighed against the risks, given the relatively benign and self-limiting nature of PFAPA.

PFAPA, like other diseases studied by consensus methodology, has no validated response measures, making comparisons between treatment arms challenging [19, 20]. Therefore, we established response criteria a priori (Table 2). Fever was chosen as the primary outcome measure. The quality of life measures were tailored to the periodic nature of PFAPA. Since PFAPA does not confer clinical sequlae (unlike FMF for example which may cause amyloidosis) and is mainly a quality of life problem, the occurrence of inflammatory marker elevation in between episodes was not considered a response criterion. Moreover, if a child with an assumed diagnosis of PFAPA has persistently elevated inflammatory markers then the diagnosis of PFAPA is incorrect.

Current therapeutic studies in PFAPA are limited by small, heterogeneous patient populations, variable diagnostic criteria, retrospective data collection, and treatment analysis that did not include comparator arms between therapeutic options [30]. Our prior survey showed considerable variation in treatment of PFAPA, both between and within subspecialties [18]. Through consensus methodology, we developed standardized regimens that will be studied in future comparative effectiveness studies.

As is the case for other CTPs, they are not intended to be guidelines or recommendations; they merely reflect current management practices. They are also not intended to capture all patients with PFAPA such as those treated with 2 or more arms at the same time or with those that have failed earlier therapies. Other limitations include the lack of validated response criteria, disease monitoring scoring tools and criteria for remission. Furthermore, we did not include all possible therapies and dosing to reduce variability. Even though treatment related morbidity can be monitored; CTPs are not primarily intended to collect information on medication safety. Finally, corticosteroids, cimetidine and colchicine have not received FDA approval for PFAPA.

Future goals are to establish evidence-based management guidelines based on the collection of prospective data from comparative effectiveness trials after implementation of the CTPs, and to identify predictors for response to specific treatments through analysis of patient characteristics in the different treatment arms. The natural history of PFAPA is resolution with age. For the anti-pyretic and corticosteroid arms, since treatment is only with episodes, as they decrease and finally stop, so does treatment. For the prophylactic arms of cimetidine and colchicine, there are no current recommendations on duration of treatment as there is insufficient data.

## Conclusion

The CARRA PFAPA group developed a four-arm CTP for PFAPA intended to be utilized in a future pilot study. Direct comparison of outcomes in each treatment arm will help identify treatments with the optimum clinical responses for this poorly understood disease.

## Supplementary information

**Additional file 1.**

## Data Availability

The datasets analyzed during the current study available from the corresponding author on reasonable request.

## Abbreviations

PFAPA: Periodic Fever, Aphthous Stomatitis, Pharyngitis and Adenitis Syndrome
CARRA: Childhood Arthritis and Rheumatology Research Alliance
CTPs: Consensus treatment plans
NGT: Nominal group technique
CEBM: Centre for Evidence-Based Medicine
PIDS: Pediatric Infectious Disease Society
