# Supplementary material for: Consensus treatment plans for periodic fever, aphthous stomatitis, pharyngitis and adenitis syndrome (PFAPA): a framework to evaluate treatment responses from the childhood arthritis and rheumatology research alliance (CARRA) PFAPA work group
Source: Pediatr Rheumatol Online J. 2020 Apr 15;18:31. doi: 10.1186/s12969-020-00424-x (PMC7157990; doi:10.1186/s12969-020-00424-x)
Supplement: Supplementary file 1 — Additional file 1. [file 12969_2020_424_MOESM1_ESM.docx]

Supplementary Table 1:

| **Number** | **First Author** | **Title** | **Journal and Year** | **Grading*** |
| --- | --- | --- | --- | --- |
| **1** | Mehregan FF | Periodic Fever, Aphthous Stomatitis, Pharyngitis and Cervical Adenitis (PFAPA) Syndrome in Iranian Children First Report of Iranian Periodic Fever and Autoinflammatory Registry (IPFAIR) | Iran J Pediatr. 2014 | 4 |
| **2** | Burton MJ | Tonsillectomy for periodic fever, aphthous stomatitis, pharyngitis and cervical adenitis syndrome (PFAPA). | Cochrane Database Syst Rev. 2014 | 1 |
| **3** | Stagi S | Vitamin D levels and effects of vitamin D replacement in children with periodic fever, aphthous stomatitis, pharyngitis, and cervical adenitis (PFAPA) syndrome. | Int J Pediatr Otorhinolaryngol. 2014 | 4 |
| **4** | Vigo G | Tonsillectomy efficacy in children with PFAPA syndrome is comparable to the standard medical treatment: a long-term observational study. | Clin Exp Rheumatol. 2014 | 4 |
| **5** | Król P | PFAPA syndrome: clinical characteristics and treatment outcomes in a large single-centre cohort. | Clin Exp Rheumatol. 2013 | 3 |
| **6** | Licameli G | Long-term surgical outcomes of adenotonsillectomy for PFAPA syndrome. | Arch Otolaryngol Head Neck Surg. 2012 | 3 |
| **7** | Yazgan H | Comparison of conventional and low dose steroid in the treatment of PFAPA syndrome: preliminary study. | Int J Pediatr Otorhinolaryngol. 2012 | 2 |
| **8** | Stojanov S | Periodic fever, aphthous stomatitis, pharyngitis, and adenitis (PFAPA) is a disorder of innate immunity and Th1 activation responsive to IL-1 blockade. | Proc Natl Acad Sci U S A. 2011 | 4 |
| **9** | Wurster VM | Long-term follow-up of children with periodic fever, aphthous stomatitis, pharyngitis, and cervical adenitis syndrome. | J Pediatr. 2011 | 4 |
| **10** | Peridis S | PFAPA syndrome in children: A meta-analysis on surgical versus medical treatment. | Int J Pediatr Otorhinolaryngol. 2010 | 1 |
| **11** | Feder HM | A clinical review of 105 patients with PFAPA (a periodic fever syndrome). | Acta Paediatr. 2010 | 4 |
| **12** | Pignataro L | Outcome of tonsillectomy in selected patients with PFAPA syndrome. | Arch Otolaryngol Head Neck Surg. 2009 | 3 |
| **13** | Wong KK | Role of Tonsillectomy in PFAPA Syndrome. | Arch Otolaryngol Head Neck Surg. 2008 | 4 |
| **14** | Licameli G | Effect of adenotonsillectomy in PFAPA syndrome. | Arch Otolaryngol Head Neck Surg. 2008 | 3 |
| **15** | Tasher D | Colchicine prophylaxis for frequent periodic fever, aphthous stomatitis, pharyngitis and adenitis episodes. | Acta Paediatr. 2008 | 4 |
| **16** | Renko M | A randomized, controlled trial of tonsillectomy in periodic fever, aphthous stomatitis, pharyngitis, and adenitis syndrome. | J Pediatr. 2007 | 2 |
| **17** | Tasher D | PFAPA syndrome: new clinical aspects disclosed. | Arch Dis Child. 2006 | 4 |
| **18** | Parikh SR | Utility of tonsillectomy in 2 patients with the syndrome of periodic fever, aphthous stomatitis, pharyngitis, and cervical adenitis. | Arch Otolaryngol Head Neck Surg. 2003 | 4 |
| **19** | Berlucchi M | Update on treatment of Marshall's syndrome (PFAPA syndrome): report of five cases with review of the literature | Ann Otol Rhinol Laryngol. 2003 | 4 |
| **20** | Galanakis E | PFAPA syndrome in children evaluated for tonsillectomy | Arch Dis Child. 2002 | 4 |
| **21** | Dahn KA | Periodic fever and pharyngitis in young children: a new disease for the otolaryngologist? | Arch Otolaryngol Head Neck Surg. 2000 Sep | 4 |
| **22** | Padeh S | Periodic fever, aphthous stomatitis, pharyngitis, and adenopathy syndrome: Clinical characteristics and outcome. | J Pediatr 1999 | 4 |
| **23** | Thomas KT | Periodic fever syndrome in children. | J. Pediatr 1999 | 4 |
| **24** | Feder HM | Cimetidine treatment for periodic fever associated with aphthous stomatitis, pharyngitis and cervical adenitis. | Pediatr Infect Dis J. 1992 | 4 |
| **25** | Marshall GS | Syndrome of periodic fever, pharyngitis, and aphthous stomatitis | J Pediatr 1987 | 4 |

* Grading was performed according to the OCEBM Levels of Evidence Working Group. “The Oxford Levels of Evidence 2”. Oxford Centre for Evidence-Based Medicine.  <https://www.cebm.net/index.aspx?o=5653>.
